# Affordability trade-offs following a public option: learning from the Colorado Option

**DOI:** 10.1093/haschl/qxaf160

**Published:** 2025-08-28

**Authors:** Andrew Shermeyer

**Affiliations:** Division of Health Policy and Management, University of Minnesota, Minneapolis, MN 55455, United States

**Keywords:** public options, marketplace, Colorado Option, premium spread

## Abstract

**Introduction:**

In 2023, Colorado implemented a public option, called the Colorado Option, and required all insurers in its Affordable Care Act (ACA) Marketplace to offer plans following a uniform benefit design. While the Colorado Option aimed to lower the cost of Marketplace coverage, it is unclear whether the policy has had its intended effect. In this study, I examine how the affordability of Marketplace coverage changed after the Colorado Option for both subsidized and unsubsidized enrollees.

**Methods:**

This descriptive analysis used the HIX Compare Individual Market datasets from 2020 to 2025 to measure changes in benchmark Silver plan premiums and premium spreads (the difference in premium between the benchmark Silver plan and lowest-premium plan) in Colorado following the Colorado Option.

**Results:**

Between 2020 and 2025, benchmark Silver plan premiums in Colorado increased by $295.84 while premium spread increased by $79.53. These increases were greater than in comparison states.

**Conclusion:**

In the years following the Colorado Option, Marketplace coverage in Colorado became more affordable for subsidized enrollees but less affordable for unsubsidized ones. States considering public options should weigh this potential trade-off as they design and implement them to make sure the public option aligns with their policy goals.

## Introduction

States are increasingly looking inward for ways to make health care more affordable and accessible as federal efforts to reform health care have stalled. One alternative that has gained popularity has been the public option—a modality of privatized public insurance, characterized by state government involvement in the benefit design and/or reimbursement rate setting of health plans sold by private carriers.^1-3^ By leveraging states’ purchasing power, public options aim to offer individuals more choice for health insurance to increase affordability and expand availability of health insurance. Currently, only Washington and Colorado have implemented a public option, but both Nevada and Minnesota have passed legislation to offer public option plans on their Affordable Care Act (ACA) Marketplaces.^4,5^ Moreover, at least 15 other states have discussed implementing public options.^6^

Previous analyses of public options used premiums to measure the affordability of coverage.^7,8^ This overlooks a key feature of Marketplace coverage: the subsidized price paid by consumers is often quite lower than the premium. Currently, approximately 92% of all Marketplace enrollees qualify for Premium Tax Credits (PTCs), which they may apply to any plan in their Marketplace to reduce their out-of-pocket premium.^9^ In Colorado specifically, 80% of Marketplace enrollees received PTCs in 2025.^10^ The size of the PTC is calculated by taking the difference between the second least-expensive Silver plan, known as a benchmark plan, and an enrollee's monthly expected premium contribution (EPC). An enrollee's EPC is calculated using a sliding scale based on their modified adjusted gross income relative to the federal poverty level. Under this structure, it is possible to calculate an enrollee's minimum cost of subsidized coverage by subtracting the premium difference between the benchmark Silver and lowest premium plan from their EPC. This premium difference between the benchmark Silver plan and lowest premium plan is called premium spread.^11,12^

While pre-subsidy premiums are a valid measure of the affordability of unsubsidized Marketplace coverage, premium spread is a better measure of the out-of-pocket premiums for subsidized Marketplace enrollees. As premium spread increases, the minimum cost of subsidized coverage decreases. Moreover, large premium spreads indicate that more enrollees can purchase low- or even zero-cost coverage.

**Figure 1.**
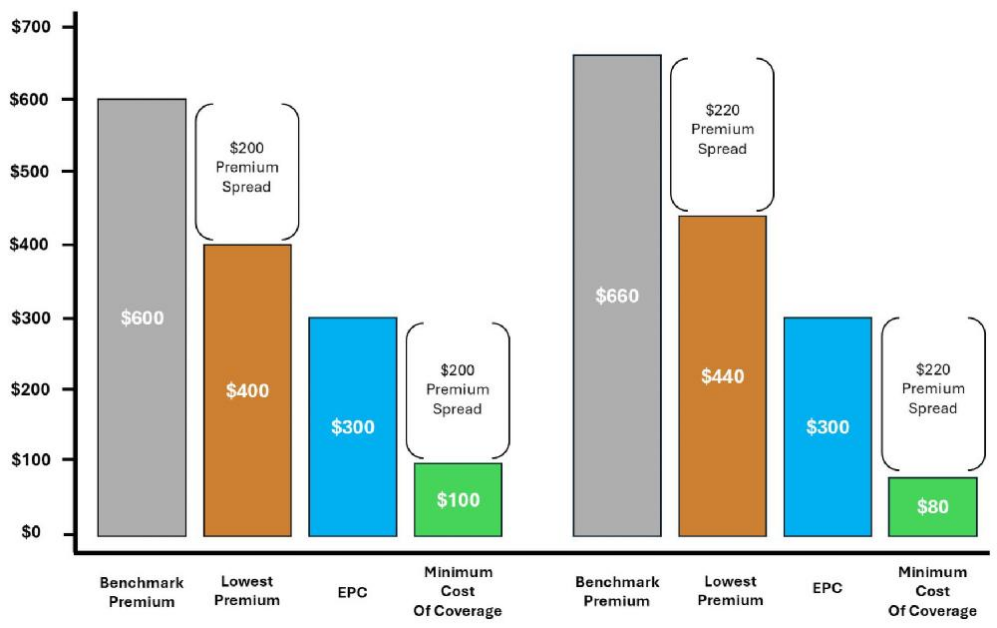
The effect of premium spread on minimum cost of coverage. Abbreviation: EPC, expected premium contribution.

To illustrate this, take, for example, a hypothetical enrollee who has an EPC of $300 in a county where the benchmark Silver plan is $600 and the minimum cost plan is $400. In this case, the premium spread is $200 ($600–$400), and the minimum cost of coverage would be the $300 EPC minus the $200 premium spread, or $100. Now, imagine that premiums increased by 10%. The EPC remains at $300 but the benchmark Silver plan is now $660 and the minimum plan is now $440. As such, the premium spread would be $220 and the minimum cost of coverage becomes $80 ($300–$220). In this example, the pre-subsidy premium increased but the post-subsidy premium decreased. If the dollar-amount increase in benchmark Silver plan premium is greater than the dollar-amount increase in the lowest premium, the premium spread will increase, and subsidized coverage will become more affordable. This example is illustrated in Figure 1.

As more states begin to design and implement public options, it is crucial that they understand the potential effects this type of policy could have on all enrollees. To date, no study has measured the effect of public options on both unsubsidized and subsidized enrollees. In this study, I analyze changes in Silver plan premiums and premium spreads across all counties in Colorado to characterize the impact that the Colorado Option has had on the affordability of Marketplace coverage.

## Data and methods

### Setting

In 2023, Colorado required all insurers participating in the state's ACA Marketplace to offer plans with uniform benefit designs in compliance with HB21-1232, commonly referred to as the “Colorado Option.”^13,14^ Through these plans, the Colorado Option intended to simultaneously increase consumer access to high-quality care while controlling premiums. Specifically, premiums were to be held to premium reduction targets based on the average premium rates that insurers offered individual plans in 2021 in each county.^13,15^ In 2023, the first year of the Colorado Option's implementation, the premium reduction target was set to 5%, with subsequent 5% reduction targets in both 2024 and 2025 (to a cumulative 15% after 3 years).^13^ From 2026 forward, Colorado Option premiums are not to increase more than medical inflation.^13^ Despite insurers frequently missing these premium reduction targets, enrollment in Colorado Option plans has increased in each year they have been available. In 2023, approximately 35 000 individuals enrolled in a Colorado Option plan, making up 13% of all Marketplace enrollments during the open enrollment period.^16^ As of 2025, this has increased to 132 791 individuals choosing a Colorado Option plan, making up 47% of all Marketplace enrollments.^10^

### Data

The primary data sources were the HIX Compare Individual Market datasets from 2020 to 2025.^17^ The Individual Market datasets include plan-level data for nearly every health plan offered to individuals on the ACA Marketplaces in all 50 states as well as the District of Columbia. Approximately 70% of the data comes directly from insurers themselves, while the remaining data are obtained through the Centers for Medicare and Medicaid Services’ public use files or the National Association of Insurance Commissioners’ System for Electronic Rate and Form Filing. The data contain information on benefits, pricing, and availability of the plans at the rating area level. The HIX Compare data also include annual crosswalk files that map counties to rating areas and identify insurer participation by county, in case they did not offer plans across entire rating areas. I adjusted pricing information for inflation relative to 2020.

### Study design and sample

This was a descriptive analysis of how benchmark Silver plan premiums and premium spreads changed in Colorado following the Colorado Option. Both Colorado Option plans and non–Colorado Option plans are included in the analysis. I compared these changes with those observed in a set of states similar to Colorado. These states were Delaware, Maryland, Minnesota, Montana, North Dakota, Rhode Island, and Wisconsin. This group has been used in a previous estimation of the Colorado Option's impact on premiums.^8^ Like Colorado, these states maintained a reinsurance program throughout the years of this analysis, 2020 to 2025.

The final sample included 2184 county-years, containing 96 313 Individual and Family Marketplace plans. All the plans were offered on an ACA Marketplace. Plans that were either a cost-sharing variant of an existing Silver plan or categorized as “catastrophic” were excluded.

### Measures

I measured benchmark Silver plan premiums and premium spreads at the county-year level. I used premiums based on the following parameters used by the HIX Compare data: 50 years of age, nonsmoking, and individual coverage. The benchmark Silver plan premium was measured as a continuous variable with a mean of $683.29 (SD = $157.37). Benchmark Silver plan premium values ranged from $383.95 (lowest) to $1138.29 (highest). I calculated premium spread by taking the difference in premium between the benchmark Silver plan and the cheapest plan in each county. Premium spread was measured as a continuous variable with a mean of $184.32 (SD = $79.42). The minimum value of premium spread was $37.52 and the maximum value was $504.21.

### Statistical analysis

I calculated the benchmark Silver plan premium and premium spread for each county between the years 2020 and 2025. I calculated the mean and SD of these measures at the state level for each year. I used R, version 4.4.1, to conduct the analysis.

### Limitations

I acknowledge a number of limitations. First, the results should not be interpreted causally. While I did run a difference-in-differences analysis of premiums and premium spread, it did not meet the parallel trends assumption and has limited interpretability. Those results are reported in Table S1. Second, my analysis only considered 1 level of premium, so the findings may not be applicable to every Marketplace enrollee. Third, my analysis may be limited in its generalizability since it only considers a single public option in a single state. Public options are flexible policies so states can design them to meet their specific needs, making exact comparisons more difficult. Fourth, while my study considered the affordability of obtaining health insurance through the Marketplace, it could not account for the cost of using health services. Future work should consider the Colorado Option's impact on the access to and utilization of health services.

## Results

**Figure 2.**
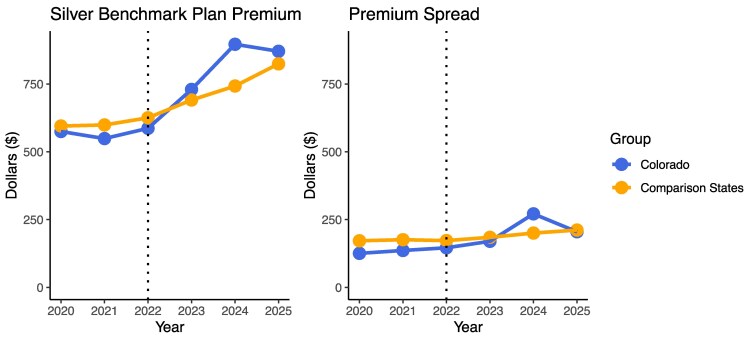
Trends in Silver benchmark plan premiums and premium spread.

Figure 2 shows how the benchmark Silver premium and premium spread changed in Colorado as well as the comparison states. In Colorado, the average benchmark Silver plan premium increased by $295.84, from $574.75 (SD = $51.41) in 2020 to $870.59 (SD = $69.56) in 2025, but peaked at $896.35 (SD = $129.87) in 2024. In comparison states, the average benchmark Silver plan premium increased by $227.83, from $592.74 (SD = $151.53) in 2020 to $820.57 (SD = $152.48) in 2025.

In Colorado, the average premium spread increased by $79.53, from $125.62 (SD = $11.00) in 2020 to $205.15 (SD = $36.89) in 2024, with a spike in 2024 at $271.20 (SD = $68.41). In comparison states, the average premium spread increased by $39.07, from $169.11 (SD = $104.79) in 2020 to $208.18 (SD = $77.43) in 2025.

## Discussion

My results show that both benchmark Silver plan premiums and premium spread increased in Colorado following the Colorado Option, suggesting that coverage became more affordable for subsidized enrollees while becoming less so for unsubsidized enrollees. These changes were larger than those seen in comparison states. My analysis suggests that the widening premium spread under the Colorado Option was primarily driven by increases in benchmark Silver plan premiums rather than reductions in the lowest-cost premiums.

Colorado's increase in premiums may also be related to other market factors such as insurer participation. One insurer, Friday Health Plans, stopped operating in Colorado midway through 2023. Notably, Friday Health Plans did not operate in any of the comparison states, so they were unaffected by their insolvency. This exit could have led to less competition and, in turn, could have contributed to the increase in premiums.^18-20^ More detailed analysis is needed to understand the relationship between the Colorado Option and insurer participation as well as its impact on market stability.

My findings differ from previous analyses of the Colorado Option and premium trends. Murray and Whaley^8^ reported a $101 decrease in minimum and benchmark Silver plan premiums relative to comparison states between 2020 and 2024, while the Urban Institute^21^ reported that average benchmark Silver plan premiums did not change between 2022 and 2023. Both studies analyzed data at the rating area level, whereas mine considered premiums at the county level. Like Murray and Whaley, my analysis found evidence of improvements to affordability, but I attribute these changes to increased premium spreads since I found that premiums themselves increased. Unlike the Urban Institute, I did not use population weights to adjust premiums. However, Figure S1 shows that both benchmark Silver premiums and premium spread still increased in Colorado when such adjustments are applied.

While this study focused specifically on the Colorado Option, its findings have broader implications for other states implementing a public option. For instance, depending on its design, a public option may introduce a trade-off of making Marketplace coverage more affordable for subsidized enrollees while making it less affordable for unsubsidized ones. In the case of Colorado, premium spread increased because the benchmark Silver plan premiums increased more than the lowest cost plan premium did. As a result, approximately 80% of Marketplace enrollees—approximately 225 000 individuals—who qualified for subsidized coverage were able to purchase Gold plans at a lower out-of-pocket premium or even Bronze plans for no out-of-pocket premium, while the remaining subsidy-ineligible individuals faced higher premiums for the same level of coverage as before.^10^

Up to this point, this trade-off has been mitigated by the enhanced subsidies provided under the American Rescue Plan Act (ARPA), which increased subsidy amounts and expanded eligibility for these subsidies.^9,22^ However, these subsidies are scheduled to expire at the end of 2025. Once the subsidies expire, fewer individuals will receive subsidies and those who do will get a smaller amount. Most individuals who purchase a plan through a Marketplace will face higher out-of-pocket costs to maintain coverage. The expiration of the ARPA-enhanced subsidies could reestablish a “subsidy cliff” where people with incomes at 400% the federal poverty level or above no longer qualify for subsidies in the Marketplace.^23^ In Colorado, this means that a smaller proportion of enrollees in the Marketplace will benefit from the widened premium spread and that more enrollees will face higher out-of-pocket premiums.

States implementing a public option must also bear in mind that insurer participation in a public option is not the same as insurer compliance with that public option. Moreover, the design of a public option could unintentionally increase the tension between the 2. To guarantee insurer participation in the public option, Colorado required insurers to offer Colorado Option plans in each county and at each metal level they offered non–Colorado Option Marketplace plans. To guarantee premium savings, Colorado required that insurers offer the new Colorado Option plans at rates set to a 2021 baseline with subsequent premium reduction targets. Most insurers, however, did not meet the required reduction targets.

In their noncompliance filings with Colorado's Division of Insurance (DOI), insurers frequently cited anticipated losses under the Colorado Option, arguing that the mandated premium reductions were actuarially unsound.^24-28^ They also pointed to the higher costs of providing enhanced essential health benefits and additional administrative burdens of the Colorado Option plans. This noncompliance prompted the DOI to schedule public rate review hearings to allow insurers and providers to testify about how specific hospitals prevented them from lowering their reimbursement rates, which, consequentially, prevented them from lowering premiums to meet the premium reduction targets.^15^ Following a hearing, the DOI Commissioner can set new reimbursement rates that providers are required to accept and from which insurers recalculate premiums. Thus, through these rate review hearings, insurers can acquire lower prices from hospitals. To date, however, no rate review hearings have taken place.

## Conclusion

Following the Colorado Option, both Silver plan premiums and premium spreads increased across the state. This indicates that Marketplace coverage became more affordable for subsidized enrollees while becoming less affordable for unsubsidized enrollees. States should consider this trade-off as they design and implement public options in the future—especially as the enhanced subsidies of the ARPA are set to expire at the end of 2025.

## Supplementary Material

qxaf160_Supplementary_Data

## Data Availability

All data are available at https://hix-compare.org/.
