# Supplementary material for: Affordability trade-offs following a public option: learning from the Colorado Option
Source: Health Aff Sch. 2025 Aug 28;3(8):qxaf160. doi: 10.1093/haschl/qxaf160 (PMC12392888; doi:10.1093/haschl/qxaf160)
Supplement: qxaf160_Supplementary_Data [file qxaf160_supplementary_data.zip › Supplemental Table 1.docx]

Supplemental Table 1: Difference-in-Differences Results

|  |  | Means |  |  | Difference-in-differences | |
| --- | --- | --- | --- | --- | --- | --- |
|  | | | | | | |
| Dependent Variable | | Pre-Colorado Option | Post-Colorado Option | Difference Between Periods | Unadjusted | Regression-adjusted |
|  |  |  |  |  |  |  |
| Benchmark Silver Plan Premium ($) | | | | | | |
|  | Colorado | 570.03 | 832.27 | 262.24 |  |  |
|  | Comparison States | 606.43 | 752.53 | 6.50 | 116.14*** | 116.14*** |
|  |  |  |  |  |  |  |
| Premium Spread ($) | | | | | | |
|  | Colorado | 135.97 | 215.46 | 79.49 |  |  |
|  | Comparison States | 173.49 | 198.82 | 25.33 | 54.15*** | 54.15*** |
|  |  |  |  |  |  |  |
|  |  |  |  |  |  |  |
|  |  |  |  |  |  |  |
| * p < 0.05 ** p < 0.01 *** p < 0.001 | | | | | | |
