# Supplementary figures and images for: Affordability trade-offs following a public option: learning from the Colorado Option
Source: Health Aff Sch. 2025 Aug 28;3(8):qxaf160. doi: 10.1093/haschl/qxaf160 (PMC12392888; doi:10.1093/haschl/qxaf160)

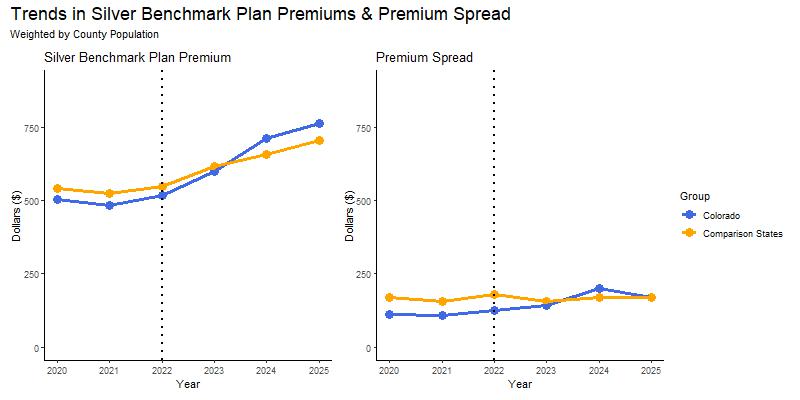

Supplement: qxaf160_Supplementary_Data [file qxaf160_supplementary_data.zip › Supplemental Figure 1.jpg]
